# Members of the *Candida parapsilosis* Complex and *Candida albicans* are Differentially Recognized by Human Peripheral Blood Mononuclear Cells

**DOI:** 10.3389/fmicb.2015.01527

**Published:** 2016-01-13

**Authors:** Eine Estrada-Mata, María J. Navarro-Arias, Luis A. Pérez-García, Erika Mellado-Mojica, Mercedes G. López, Katalin Csonka, Attila Gacser, Héctor M. Mora-Montes

**Affiliations:** ^1^Departamento de Biología, División de Ciencias Naturales y Exactas, Campus Guanajuato, Universidad de GuanajuatoGuanajuato, México; ^2^Centro de Investigacion y de Estudios Avanzados del Instituto Politécnico NacionalIrapuato, México; ^3^Department of Microbiology, University of SzegedSzeged, Hungary

**Keywords:** *Candida parapsilosis*, *Candida albicans* host-fungus interaction, cell wall, cytokine, mononuclear cells

## Abstract

The systemic infections caused by members of the *Candida parapsilosis* complex are currently associated to high morbility and mortality rates, and are considered as relevant as those caused by *Candida albicans.* Since the fungal cell wall is the first point of contact with the host cells, here we performed a comparison of this organelle in members of the *C. parapsilosis* complex, and its relevance during interaction with human peripheral blood mononuclear cells (PBMCs). We found that the wall of the *C. parapsilosis* complex members is similar in composition, but differs to that from *C. albicans*, with less mannan content and more β-glucan and porosity levels. Furthermore, lectin-based analysis showed increased chitin and β1,3-glucan exposure at the surface of *C. parapsilosis sensu lato* when compared to *C. albicans*. Yeast cells of members of the *C. parapsilosis* complex stimulated more cytokine production by human PBMCs than *C. albicans* cells; and this significantly changed upon removal of *O*-linked mannans, indicating this wall component plays a significant role in cytokine stimulation by *C. parapsilosis sensu lato*. When inner wall components were exposed on the wall surface, *C. parapsilosis sensu stricto* and *C. metapsilosis*, but not *C. orthopsilosis*, stimulated higher cytokine production. Moreover, we found a strong dependency on β1,3-glucan recognition for the members of the *C. parapsilosis* complex, but not for live *C. albicans* cells; whereas TLR4 was required for TNFα production by the three members of the complex, and stimulation of IL-6 by *C. orthopsilosis*. Mannose receptor had a significant role during TNFα and IL-1β stimulation by members of the complex. Finally, we demonstrated that purified *N*- and *O*-mannans from either *C. parapsilosis sensu lato* or *C. albicans* are capable to block the recognition of these pathogens by human PBMCs. Together; our results suggest that the innate immune recognition of the members of the *C. parapsilosis* complex is differential of that reported for *C. albicans*. In addition, we propose that purified cell wall mannans can be used as antagonist to block specific receptors on innate immune cells.

## Introduction

Fungal infections are currently a burden for most of the health systems worldwide, and among them, superficial and invasive candidiasis are of special interest, since the latter has a mortality rate higher than 45% in infected patients (Brown et al., 2012). *Candida albicans* is the most frequent causative agent of candidiasis, being responsible of about 50% of total invasive candidiasis, while other members of the *Candida* genus, named emerging species, contribute together to the rest of the reported cases (Trofa et al., 2008). *Candida parapsilosis sensu lato* is a species that is mostly found in neonate patients, causing more than 33% of invasive candidiasis in this group (Pammi et al., 2013). It is a versatile yeast-like organism that, at difference of other pathogenic *Candida* species, can be found colonizing non-human organisms and inert material from the environment (Trofa et al., 2008). This organism is in fact a complex of three closely related species: *C. parapsilosis sensu stricto*, *Candida orthopsilosis* and *Candida metapsilosis* (Tavanti et al., 2005); which have subtle, but key differences in terms of virulence (Nemeth et al., 2013; Gago et al., 2014), drug sensitivity (Spreghini et al., 2012; Szenzenstein et al., 2013), and secretion of hydrolytic enzymes (Trevino-Rangel Rde et al., 2013).

The establishment of a protective anti-*Candida* immune response in the host relays on a proper activation of the innate immune branch, and significant efforts have been done to understand this host–pathogen interaction, using *C. albicans* as a model (Netea et al., 2015). In *C. parapsilosis sensu lato*, there is currently limited information about the mechanisms underlying its interaction with components of the innate immunity. Thus far, it has been demonstrated that galectin 3 expressed by human neutrophils drives an increased *C. parapsilosis* phagocytosis, but not when challenged against *C. albicans* yeast cells (Linden et al., 2013). In addition, human peripheral blood mononuclear cells (PBMCs) stimulated with heat-killed (HK) *C. parapsilosis sensu stricto* yeast cells produced lower Interleukin (IL) 1β, interferon γ, IL-17 and IL-22, but higher levels of IL-10, when compared to cells confronted with *C. albicans* (Toth et al., 2013). Despite this progress, there are not reports dealing with the interaction of immune cells with members of the *C. parapsilosis* complex.

The fungal cell wall contains most of the pathogen-associated molecular patterns recognized by pattern recognition receptors (PRRs) on innate immune cells, and again, the *C. albicans* cell wall is the best model thus far characterized (Díaz-Jiménez et al., 2012). This structure is composed of four main polysaccharides arranged in two well defined layers: the outermost layer composed of glycoproteins, bearing *N*- and *O*-linked mannans, and the components of the inner layer, chitin, β1,6- and β1,3-glucans (Díaz-Jiménez et al., 2012). The participation of this organelle in the activation of the innate immune response has been thoroughly studied. It is now well established that β1,3-glucan is normally hidden from the recognition of dectin-1 and TLR2, and if accessible, plays a major role in the induction of pro-inflammatory cytokines and phagocytosis by macrophages (Gantner et al., 2005; Gow et al., 2007; Heinsbroek et al., 2008). The *N*-linked mannans play also a significant role in both cytokine stimulation and macrophage uptake, via the mannose receptor (MR), dectin-2, and DC-SIGN (Netea et al., 2006; Mora-Montes et al., 2007, 2010; Cambi et al., 2008; McKenzie et al., 2010; Saijo and Iwakura, 2011). On the contrary, *O*-linked mannans play a dispensable role in both immunological processes, although it engages to TLR4 (Netea et al., 2006), a potent PRRs that plays a significant role in controlling bacteria (Miyake, 2007). Thus far, there is limited information about the cell wall of members of the *C. parapsilosis* complex, and the particular contribution of PRRs in the activation of cytokine production. Here, we performed a comparative study of the cell wall composition of *C. albicans*, *C. parapsilosis sensu stricto*, *C. orthopsilosis*, and *C. metapsilosis* and found that, although the composition is similar, the arrangement of the components has significant differences that impact their ability to activate human PBMCs. Moreover, we demonstrated that purified *N*- and *O*-linked mannans from either *C. albicans*, *C. parapsilosis sensu stricto*, *C. orthopsilosis* or *C. metapsilosis* are capable to block the recognitions of these pathogens by human PBMCs.

## Materials and Methods

### Strains and Culturing Conditions

*Candida albicans* SC5314 (Gillum et al., 1984), *C. parapsilosis sensu stricto* SZMC 8110, *C. orthopsilosis* SZMC 1545, and *C. metapsilosis* SZMC 1548 (Szenzenstein et al., 2013) were used in this study. Cells were propagated at 30°C in Sabouraud broth [1% (w/v) mycological peptone, 4% (w/v) glucose], and maintained in plates containing medium added with 2% (w/v) agar. For all the experiments here reported, 500 μL of overnight-grown cells were used to inoculated 100 mL of fresh medium and incubated at 30°C with shaking at 200 rpm, until reach the mid-log growth phase (typically 5–6 h). Cells were incubated at 56°C for 1 h for heat inactivation as reported (Mora-Montes et al., 2007). For all the cases, inactivation was confirmed by loss of fungal growth in Sabouraud medium at 30°C for 72 h. To remove *O*-linked mannans, cells were incubated overnight with 100 mM NaOH as described (Diaz-Jimenez et al., 2012). Under these conditions, more than 94% cells kept viability, as tested by CFU/mL before and after treatment with NaOH.

### Cell Wall Analysis

Cell homogenates were obtained in a Braun homogenizer, with 5 cycles of 30 s and cooling on ice in-between (Mora-Montes et al., 2007). The cell walls were pelleted by centrifugation and thoroughly washed with deionized water. Further wall cleansing was performed with hot 2% (v/v) SDS, 0.3 M β-mercaptoethanol, and 1 M NaCl as described (Mora-Montes et al., 2007). Freeze-dried cell walls were hydrolyzed by adding 2 M trifluoroacetic acid and boiling for 3 h; then, the acid was evaporated, and samples were suspended in deionized water. The hydrolysates were analyzed by High Performance Anion Exchange Chromatography coupled to Pulsed Amperometric Detection (HPAEC-PAD), using a gradient of sodium acetate in 150 mM NaOH (flow 0.5 mL/min) as follows: 0–5 min = 45–75 mM NaOH, 5.1–15.0 min = 90 mM NaOH, 15.1–17.0 min = 105 mM NaOH + 75 mM sodium acetate, 17.1–20.0 min = 75 mM NaOH + 150 mM sodium acetate, and 20.1–25.0 min = 45 mM NaOH, at a column temperature of 25°C. Applied potentials for detection by the amperometric pulse were: E1 (400 ms), E2 (20 ms), E3 (20 ms), and E4 (60 ms) of +0.1, -2.0, +0.6, and -0.1 V, respectively. Protein content was determined upon lyophilized cell walls were alkali-hydrolyzed (Mora-Montes et al., 2007), using the Bradford protein assay.

The cell wall porosity was assessed using the polycation method previously reported (De Nobel et al., 1990). Aliquots containing 1 × 10^8^ cells were pelleted, the supernatant discarded and cells suspended in either 10 mM Tris-HCl, pH 7.4 (buffer A), buffer A plus 30 μg/mL poly-L-lysine (MW 30–70 kDa, Sigma Cat. No. P-2636) or buffer A plus 30 μg/mL DEAE-dextran (MW 500 kDa, Sigma Cat. No. D-9885). Then, cells were incubated at 30°C for 30 min, and shaking (200 rpm), pelleted by centrifuging, the supernatant saved, and further centrifuged before reading the absorbance at 260 nm. The relative cell wall porosity to DEAE-dextran was calculated as described (De Nobel et al., 1990).

The level of cell wall phosphomannan was determined by the ability of cells to bind the cationic dye Alcian Blue as described (Hobson et al., 2004).

### Analysis of Chitin and β1,3-Glucan Exposure at the Cell Surface

Cells were labeled with 1 mg/mL fluorescein isothiocyanate-wheat germ agglutinin conjugate (WGA-FITC; Sigma) (Mora-Montes et al., 2011) for chitin staining; while β1,3-glucan was labeled with 5 μg/mL IgG Fc-Dectin-1 chimera (Graham et al., 2006) for 40 min at room temperature, followed by incubating with 1 μg/mL donkey anti Fc IgG-FITC for 40 min at room temperature (Marakalala et al., 2013). In both cases, samples were examined by fluorescence microscopy using a Zeiss Axioscope-40 microscope and an Axiocam MRc camera. Pixels associated to 100 fluorescent cells were obtained with Adobe Photoshop^TM^ CS6 and the following formula: [(total of green pixels-background green pixels) × 100]/total pixels.

### Ethics Statement

Universidad de Guanajuato, though the Ethics Committee, approved the use of human cells in this study (permission number 17082011). Blood samples from healthy adult volunteers were obtained upon information about the study was provided and written informed consent was signed.

### Human PBMCs-*Candida* Interaction

Human PBMCs were isolated by density centrifugation using Histopaque-1077 (Sigma) as reported (Endres et al., 1988). The immune cell-fungus interaction was performed in 96-well microplates with 5 × 10^5^ PBMCs, in 100-μL RPMI 1640 Dutch modification (Sigma), and 100 μL with 1 × 10^5^ fungal cells. When required, PBMCs were pre-incubated for 60 min at 37°C with either 200 μg/mL purified mannan, laminarin (200 μg/mL), anti-MR (10 μg/mL, Invitrogen, Cat. No. Mab-Hmr) or anti-TLR4 (10 μg/mL, Santa Cruz Biotechnology, Cat. No. sc-293072) prior to stimulation with yeast cells. Isotype matched, irrelevant IgG_1_ antibodies (10 μg/mL, Santa Cruz Biotechnology, Cat. No. sc-52003) were used as controls for experiments assessing MR and TLR4. Despite all the reagents used for the pre-incubation experiments were negative to contamination by LPS (tested with the *Limulus* amebocyte lysate from Sigma) all reactions were performed in presence of 5 μg/mL polymyxin B (Sigma) (Schwartz et al., 1972). In all cases, the interactions were incubated for 24 h at 37°C with 5% (v/v) CO_2_, plates were centrifuged for 10 min at 3000 × *g* and 4°C and supernatants saved and kept at -20°C until used. ELISA kits from Peprotech were used to measure the concentration of TNFα, IL-6, and IL-10; while IL-1β levels were measured using an ELISA kit from R&D Systems. Mock interactions with only human PBMCs incubated with RPMI 1640 Dutch modification were included as negative controls. For all the cases, the amount of cytokine quantified in the negative controls was subtracted before data analysis.

### *N*- and *O*-Linked Mannan Isolation

The *N*- and *O*-linked mannans were isolated, and purified using the endoglycosidase H and β-elimination strategy previously reported by our group (Mora-Montes et al., 2012). Absence of protein and other wall polysaccharides was confirmed by the Bradford method and HPAEC-PAD, respectively (Mora-Montes et al., 2012); while lack of bacterial and fungal contamination was assessed by negative detection of LPS and no growth in LB and Sabouraud broth for 72 h at 37 and 28°C, respectively.

### Statistical Analysis

Statistical analysis was performed using GraphPad Prism 6 software. Cytokine stimulation using human PMBCs was performed in duplicate with six healthy donors, whereas the rest of the experiments were performed at least thrice in duplicate. Data represent cumulative results of all experiments performed and are showed as mean ± SD. The Mann–Whitney *U* test or unpaired *t*-test was used to establish statistical significance (see figure legends for details), with a significance level set at *P* < 0.05.

## Results

### Members of the *C. parapsilosis* Complex have Similar Composition in the Cell Wall but Differ from that Present in *C. albicans*

**FIGURE 1:**
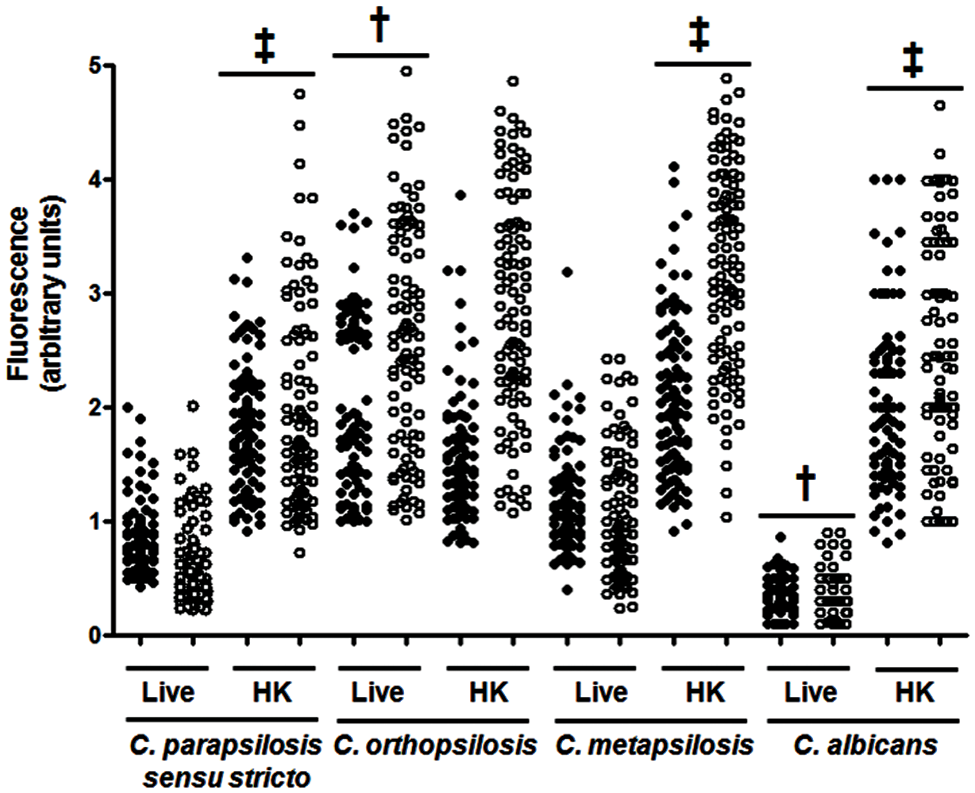
**Analysis of β1,3-glucan and chitin exposure at the cell wall surface**. Live (L) yeast cells were incubated with IgG Fc-Dectin-1and then with FITC-conjugated IgG to label β1,3-glucan (closed circles). Alternatively, chitin was labeled with WGA-FITC (open circles). The fluorescence associated to each cell is plotted. In addition, the experiment was conducted with cells previously heat-inactivated (HK). Strains used are *Candida parapsilosis sensu stricto* SZMC 8110, *C. orthopsilosis* SZMC 1545, *C. metapsilosis* SZMC 1548, and *C. albicans* SC5314. One hundred cells were analyzed for each group. ‡*P* < 0.05, when compared with the live cells; †*P* < 0.05 when compared to the other groups with live cells.

In order to assess the proportions of the main cell wall components in the three members of the *C. parapsilosis* complex, cell walls where isolated, acid-hydrolyzed and the proportion of *N*-acetylglucosamine, mannose and glucose, the basic unit of chitin, mannan and β-glucan, respectively (Mora-Montes et al., 2007, 2010, 2012), were quantified by HPAEC-PAD. *C. parapsilosis sensu stricto*, *C. orthopsilosis* and *C. metapsilosis* showed similar levels of the three polysaccharides analyzed (**Table 1**), but when compared with *C. albicans*, the species belonging to the complex showed significantly less mannan and increased β-glucan content (**Table 1**). In terms of chitin content, only *C. metapsilosis* wall displayed higher content than the *C. albicans* cell wall (**Table 1**). No differences were observed for protein content in the four yeast species analyzed (**Table 1**). Next, in order to confirm the lower mannan content in the members of the *C. parapsilosis* complex, we assessed the level of phosphomannan content and the wall porosity, parameters that suffer alterations depending on the status of the protein mannosylation pathways (De Nobel et al., 1990; Bates et al., 2005, 2006; Mora-Montes et al., 2007, 2010; West et al., 2013; Lopes-Bezerra et al., 2015). Interestingly, the cell wall from *C. parapsilosis sensu stricto*, *C. orthopsilosis* and *C. metapsilosis* showed lower phosphomannan content and higher porosity, when compared with *C. albicans* cells (**Table 1**). To assess whether the β1,3-glucan and chitin fibers in the members of the complex are underneath the mannan layer, as reported in *C. albicans* (Gow et al., 2007; Mora-Montes et al., 2011), yeast cells were labeled with the IgG Fc-Dectin-1 chimera (Graham et al., 2006), and the lectin-β1,3-glucan interaction revealed with FITC-conjugated IgG; whereas chitin was labeled with WGA-FITC. The analysis of the fluorescence associated to 100 cells indicated that, *C. parapsilosis sensu stricto*, *C. orthopsilosis*, and *C. metapsilosis* have β1,3-glucan and chitin more exposed the at the cell surface than *C. albicans* cells (**Figure 1**). However, when compared the three members of the complex, *C. orthopsilosis* displayed significantly higher levels of both polysaccharides at the wall surface than the other two species (**Figure 1**). As control, a similar experiment was conducted with HK yeast cells, where the components of the inner wall layer were artificially exposed at the cell wall surface and as consequence, higher labeling with the lectins was observed, with exception of *C. orthopsilosis*, where HK and live cells had similar content of both polysaccharides at the surface (**Figure 1**). Overall, these data indicate that the cell wall composition and organization among members of the *C. parapsilosis* complex differ to that described in *C. albicans*.

**Table 1:** Cell wall analysis of *Candida albicans* and members of the *C. parapsilosis* complex.

	Cell wall abundance				
Organism	Chitin (%)	Mannan (%)	Glucan (%)	Phosphomannan content (μg)^a^	Porosity (%)^b^	Protein (μg)^c^
*C. albicans*	2.6 ± 1.2	33.0 ± 5.0	65.0 ± 3.3	127.2 ± 9.4	28.2 ± 8.7	125.7 ± 21.1
*C. parapsilosis s. str.*	5.2 ± 2.7	13.2 ± 6.8ˆ*	81.6 ± 9.4ˆ*	72.5 ± 7.5ˆ*	60.9 ± 3.9ˆ*	143.6 ± 47.3
*C. orthopsilosis*	4.9 ± 1.4	20.5 ± 1.3ˆ*	74.6 ± 0.9ˆ*	58.5 ± 12.6ˆ*	57.2 ± 1.7ˆ*	107.0 ± 20.7
*C. metapsilosis*	6.6 ± 1.3ˆ*	21.7 ± 1.5ˆ*	71.7 ± 2.7ˆ*	85.4 ± 17.9ˆ*	71.8 ± 13.1ˆ*	97.3 ± 25.0

*^a^μg of Alcian Blue bound/OD_*600*_ = 1; ^b^Relative to DEAE-Dextran; ^c^μg of protein/mg of cell wall; ^∗^*P*< 0.05, when compared to *C. albicans.**

### *C. albicans* and *C. parapsilosis* sensu lato Differentially Stimulate Cytokine Production by Human PBMCs

**FIGURE 2:**
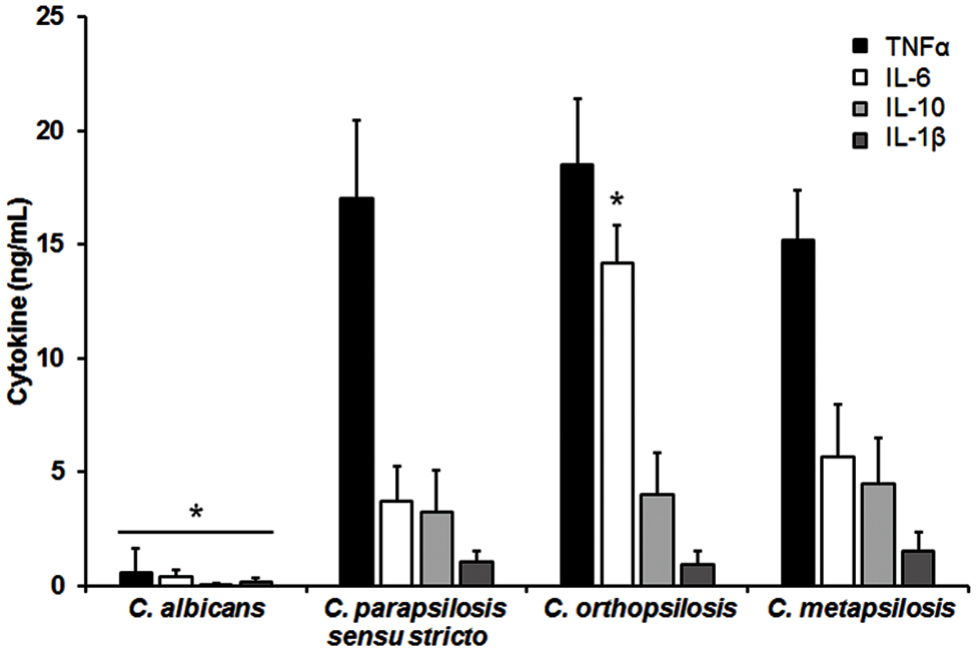
***Candida albicans* and members of the *C. parapsilosis* complex differentially stimulate the cytokine production by human PBMCs**. Human PBMCs were co-incubated 24 h with live yeast cells, the supernatant collected and used to quantify the cytokine levels. Strains used are *C. albicans* SC5314, *C. parapsilosis sensu stricto* SZMC 8110, *C. orthopsilosis* SZMC 1545, and *C. metapsilosis* SZMC 1548. ^∗^*P* < 0.05, when compared with the cytokine level stimulated by other species.

**FIGURE 3:**
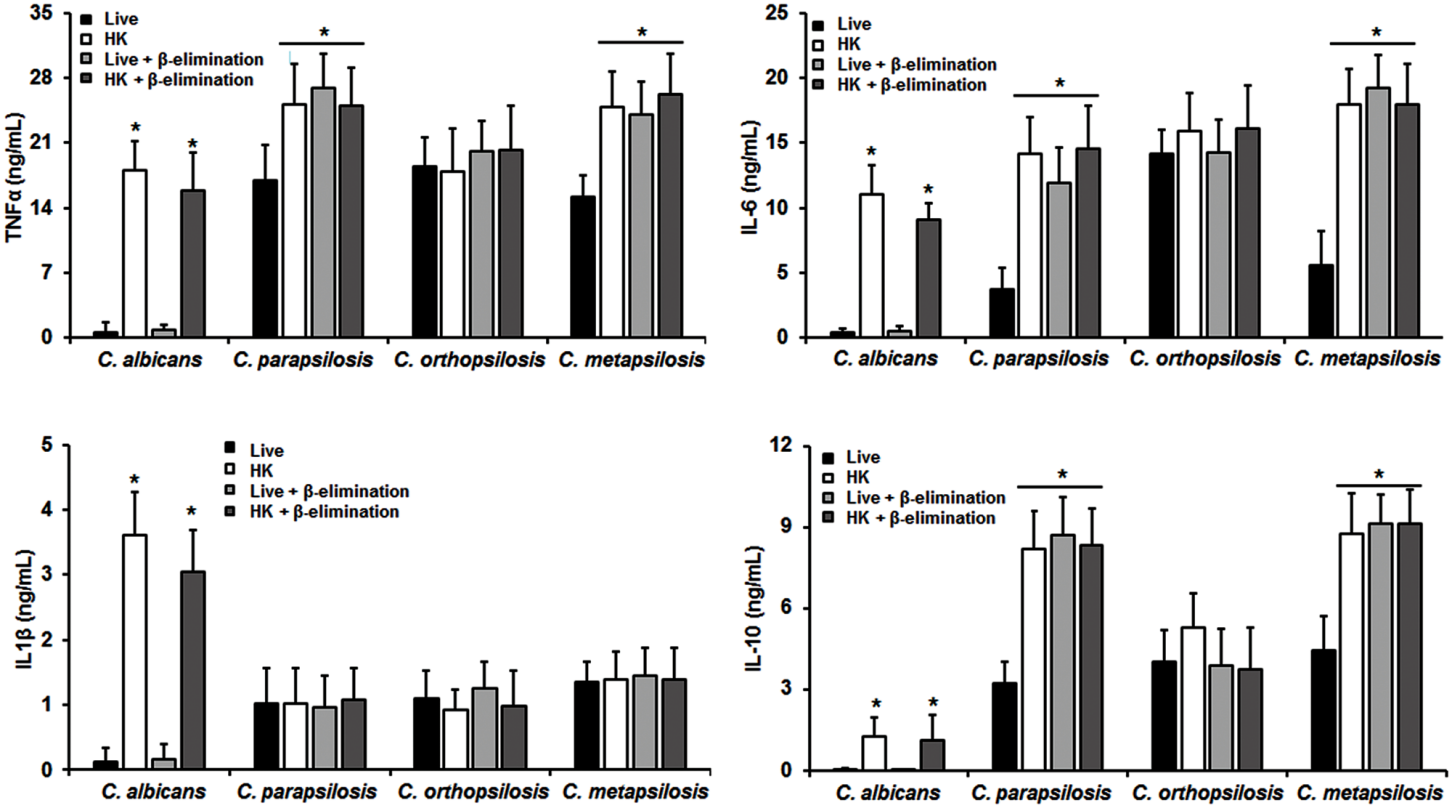
**Cytokine production by human PBMCs stimulated with either live, heat-killed (HK) or β-eliminated yeast cells**. Human PBMCs were co-incubated 24 h with either live, HK, live and β-eliminated, or HK and β-eliminated yeast cells, supernatant collected and used to quantify the cytokine levels. Strains used are *C. albicans* SC5314, *C. parapsilosis sensu stricto* SZMC 8110, *C. orthopsilosis* SZMC 1545, and *C. metapsilosis* SZMC 1548. ^∗^*P* < 0.05, when compared with live cells from the same species.

The differences in composition and organization of the cell wall above described may suggest that the fungus-immune cell interaction differs between member of the *C. parapsilosis* complex and *C. albicans*. Thus, in order to get some insights about such interaction, yeast cells were co-incubated with human PBMCs and secreted cytokines levels were measured as a read out of such interaction. Live *C. albicans* cells barely stimulated the production of either TNFα, IL-1β, IL-6 or IL-10 (**Figure 2**); however, *C. parapsilosis sensu stricto*, *C. orthopsilosis*, and *C. metapsilosis* stimulated higher and similar levels of TNFα, IL-1β, and IL-10 (**Figure 2**). Although IL-6 stimulation was significantly higher during the interaction with any of the three members of the *C. parapsilosis* complex, *C. orthopsilosis* stimulated the highest IL-6 production, when compared with the other species analyzed (**Figure 2**). Next, we compared the cytokine profile stimulated with either live or HK cells with or without β-elimination treatment to remove *O*-linked mannans from the cell wall (Diaz-Jimenez et al., 2012). As previously reported for *C. albicans* (Gow et al., 2007), HK yeast cells stimulated significant levels of TNFα, IL-1β, IL-6, and IL-10, when compared to live cells (**Figure 3**). A subtle, not significant reduction was observed in the production of the cytokines upon stimulation of human PBMCs with β-eliminated HK *C. albicans* cells (**Figure 3**). Upon heat-inactivation, *C. parapsilosis sensu stricto* and *C. metapsilosis* stimulated increased levels of TNFα, IL-6 and IL-10, and these cytokine levels were similar when live yeast cells were *O*-deglycosylated, i.e., β-eliminated (**Figure 3**). No further changes in the cytokine stimulation were observed when this treatment was applied to HK cells (**Figure 3**). For stimulation of IL-1β, modest levels of this cytokine were obtained, being not as abundant as those stimulated with HK *C. albicans* cells, as reported (Toth et al., 2013), and displaying no significant changes even though cells were both HK and β-eliminated (**Figure 3**). For *C. orthopsilosis*, the heat killing and β-elimination did not affect production of TNFα, IL-1β, IL-6 and IL-10 by human PBMCs. Therefore, *C. parapsilosis sensu lato* is capable to stimulate a different cytokine production than that observed in *C. albicans*, when incubated with human PBMCs. Furthermore, these data indicate that *C. parapsilosis sensu stricto* and *C. metapsilosis* but not *C. orthopsilosis*, have a similar interaction with these immune cells.

### Role of PRRs in the Sensing of Members of the *C. parapsilosis* Complex

**FIGURE 4:**
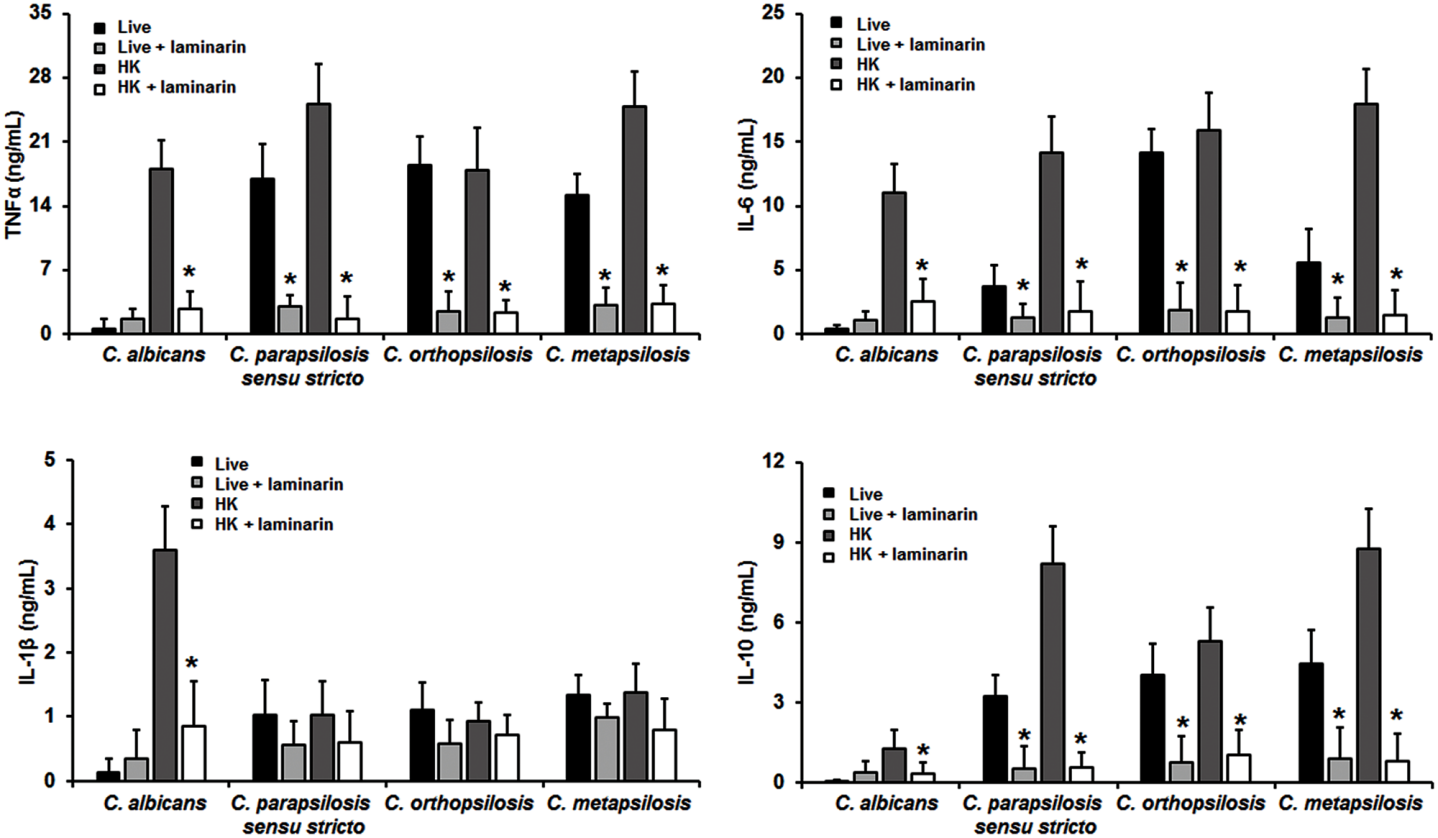
**Cytokine production by human PBMCs pre-incubated with laminarin**. Human PBMCs were pre-incubated 60 min with 200 μg/mL laminarin before challenged for 24 h with either live or HK yeast cells, supernatant were collected and used to quantify the cytokine levels. Strains used are *C. albicans* SC5314, *C. parapsilosis sensu stricto* SZMC 8110, *C. orthopsilosis* SZMC 1545, and *C. metapsilosis* SZMC 1548. ^∗^*P* < 0.05, when compared with untreated cells from the same organism.

Next, in order to assess the importance of some PRRs during the fungus-immune cell interaction, human PBMCs were pre-incubated with antagonist of specific PRRs before the interaction with fungal cells. To evaluate the relevance of dectin-1 during this interaction, human PBMCs were pre-incubated with the specific antagonist laminarin (Maneu et al., 2011; Huang et al., 2012; Cohen-Kedar et al., 2014). Results showed in **Figure 4** indicate that stimulation of TNFα, IL-1β, IL-6, and IL-10 by HK *C. albicans* cells was significantly dependent on the engagement of dectin-1 with β1,3-glucan, but not for live cells. When similar experiments were conducted with members of the *C. parapsilosis* complex, levels of TNFα, IL-6, and IL-10 stimulated with the three species were significantly reduced upon pre-incubation with laminarin, indicating a strong dependence on this receptor for their stimulation (**Figure 4**). At difference of *C. albicans*, stimulation of cytokines by live *C. parapsilosis sensu lato* cells was significantly sensitive to the presence of the dectin-1 antagonist. Interestingly, when human PBMCs were stimulated with *C. parapsilosis sensu lato* the IL-1β production was insensitive to the dectin-1 blocking, suggesting this receptor does not play a significant role for IL-1β stimulation by members of the *C. parapsilosis* complex.

**FIGURE 5:**
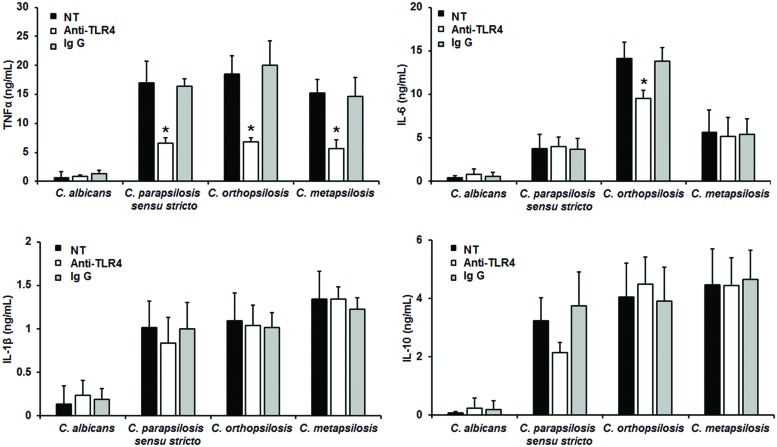
**Cytokine production by human PBMCs pre-incubated with anti-TLR4 antibodies**. Human PBMCs were pre-incubated 60 min with 10 μg/mL either anti-TLR4 antibodies or IgG_1_ before challenged for 24 h with live cells, supernatant were collected and used to quantify the cytokine levels. Strains used are *C. albicans* SC5314, *C. parapsilosis sensu stricto* SZMC 8110, *C. orthopsilosis* SZMC 1545, and *C. metapsilosis* SZMC 1548. ^∗^*P* < 0.05, when compared with untreated cells from the same organism. NT, non-treated cells.

To assess the relevance of TLR4 receptor during recognition of *C. parapsilosis sensu lato*, human PBMCs were pre-incubated with blocking anti-TLR4 antibodies before challenged with yeast cells. Results shown in **Figure 5** indicate that blocking of TLR4 has no impact on the cytokine production stimulated by live *C. albicans* cells. Similar experiments conducted with HK cells did not show significant differences in the levels of cytokine production (not shown). TNFα production stimulated by the three members of the *C. parapsilosis* complex was significantly reduced when TLR4 was blocked (**Figure 5**); while IL-6 stimulation was significantly diminished only when human PBMCs were co-incubated with *C. orthopsilosis* (**Figure 5**). Experiments conducted in presence of an irrelevant antibody of the same isotype gave no significant differences in the cytokine level (**Figure 5**). No significant changes where observed when IL-10 and IL-1β were quantified after pre-incubation with the TLR4 blocking antibodies (**Figure 5**). Therefore, TLR4 is required for TNFα production but not for IL-10 nor IL-1β, when human PBMCs are challenged with *C. parapsilosis sensu lato*. Furthermore, production of IL-6 was specifically affected when TLR4 engagement was disrupted in cells stimulated with *C. orthopsilosis*.

**FIGURE 6:**
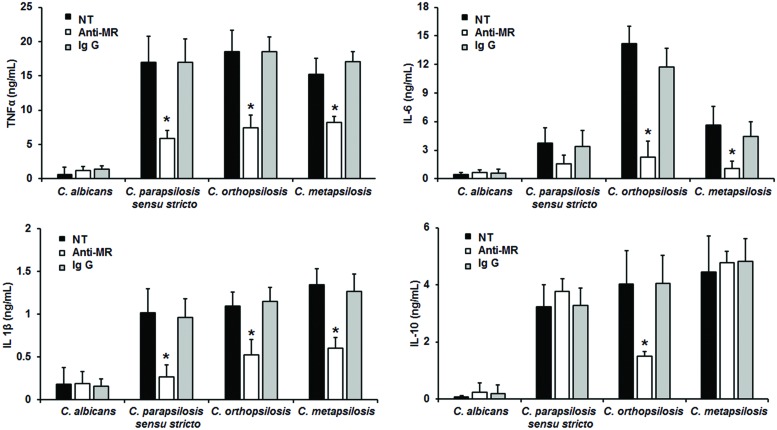
**Cytokine production by human PBMCs pre-incubated with anti-MR antibodies**. Human PBMCs were pre-incubated 60 min with 10 μg/mL either anti-MR antibodies or IgG_1_ before challenged for 24 h with live cells, supernatant were collected and used to quantify the cytokine levels. Strains used are *C. albicans* SC5314, *C. parapsilosis sensu stricto* SZMC 8110, *C. orthopsilosis* SZMC 1545, and *C. metapsilosis* SZMC 1548. ^∗^*P* < 0.05, when compared with untreated cells from the same organism. NT, non-treated cells.

When the yeast-PBMC interaction was performed with human cells pre-treated with anti-MR antibodies, there were no significant changes in the cytokine production stimulated by *C. albicans* cells (**Figure 6**). However, TNFα, and IL-1β stimulation by *C. parapsilosis sensu lato* was significantly reduced upon MR blocking (**Figure 6**). IL-6 levels were only significantly reduced in anti-MR-pre-incubated human PBMCs stimulated with either *C. orthopsilosis* or *C. metapsilosis*, but not with *C. parapsilosis sensu stricto* (**Figure 6**); while blocking of MR only affected IL-10 production in cells stimulated with *C. orthopsilosis* (**Figure 6**). Control interactions with an irrelevant antibody did not show changes in the cytokine production stimulated by all tested yeast cells (**Figure 6**). Taken together, these results indicate the role of dectin-1, TLR4 and MR in the recognition of members of the *C. parapsilosis* complex and *C. albicans* is different.

### Purified Mannans from *C. albicans* and *C. parapsilosis sensu lato* are Capable to Mimic the Effect of Anti-TLR4 and Anti-MR Antibodies on Human PMBCs

Purified cell wall components from *C. albicans* have been previously used to block its proper recognition by human PBMCs (Mora-Montes et al., 2011), thus we aimed to isolate either *N*-linked or *O*-linked mannan using well-established protocols (Mora-Montes et al., 2012). Purified mannans from any of the three members of the *C. parapsilosis* complex or *C. albicans* were negative to fungal, bacteria and protein or other wall polysaccharide contamination, and did not stimulate any cytokine production when used at concentration up to 400 μg/mL (not shown). When human PBMCs were pre-incubated with 200 μg/mL *N*- or *O*-linked mannans from *C. albicans* and then stimulated with *C. parapsilosis sensu stricto*, TNFα stimulation was significantly reduced, but not IL-6 production (**Table 2**). Similar results were observed when mannans from either *C. parapsilosis sensu lato*, *C. orthopsilosis* or *C. metapsilosis* were used in the pre-incubation step (**Table 2**), or when mannans were used to block the proper recognition of *C. orthopsilosis* or *C. metapsilosis* (not shown). Therefore, *N*- and *O*-linked mannans are able to block the recognition of *C. parapsilosis sensu lato* as the anti-MR and anti TLR4 antibodies, respectively.

**Table 2:** Blocking of cytokine production by *N*- and *O*-linked mannans isolated from either *C. albicans* or *C. parapsilosis sensu lato* cell wall.

Cytokine stimulation by *C. parapsilosis sensu stricto*	TNFα (%)	IL-6 (%)
No mannan included	100	100
Pre-incubation with *C. albicans N*-linked mannan	34.4 ± 4.3^∗^	90.4 ± 5.4
Pre-incubation with *C. albicans O*-linked mannan	43.8 ± 7.6^∗^	99.3 ± 3.2
Pre-incubation with *C. parapsilosis s. st. N*-linked mannan	39.4 ± 6.3^∗^	93.3 ± 4.3
Pre-incubation with *C. parapsilosis s. st. O*-linked mannan	40.3 ± 6.3^∗^	101.2 ± 4.3
Pre-incubation with *C. orthopsilosis N*-linked mannan	33.4 ± 9.3^∗^	101.4 ± 3.4
Pre-incubation with *C. orthopsilosis O*-linked mannan	42.3 ± 4.9^∗^	95.8 ± 4.2
Pre-incubation with *C. metapsilosis N*-linked mannan	35.6 ± 6.6^∗^	95.3 ± 5.5
Pre-incubation with *C. metapsilosis O*-linked mannan	43.8 ± 3.9^∗^	100.5 ± 6.3

*^∗^*P*< 0.05, when compared to stimulation with no mannan included.*

## Discussion

Members of the *Candida* genus are frequently regarded as similar to *C. albicans*; however, it is possible to demonstrate they have both genetic and metabolic differences that can affect the interaction with the host (Butler et al., 2009). Here, our comparative analysis of cell wall composition showed that members of the *C. parapsilosis* complex displayed lower mannan quantity, with similar protein level, when compared to *C. albicans* cells. In order to explain these data, we hypothesize that the mannans displayed on the wall surface have lower content of mannose per oligosaccharide unit, i.e., they are shorter in *C. parapsilosis sensu lato*. Indeed, it has been demonstrated *C. parapsilosis* has shorter *N*-linked mannans than those from the *C. albicans* cell wall (Shibata et al., 1995). The presence of short mannans is likely to affect the cell wall porosity, offering higher ability to DEAE-dextran, a bulky polycation, to reach the plasma membrane with the consequent increment in nucleic acid leakage (De Nobel et al., 1990). Moreover, these shorter mannans could be responsible of the increased content of both chitin and β1,3-glucan exposed at the wall surface. Our hypothesis of shorter mannans on the surface of *C. parapsilosis sensu lato* cells is also supported by the reduced phosphomannan content, which is an indirect measurement of both *N*- and *O*-linked mannan length (Bates et al., 2005, 2006; Mora-Montes et al., 2007, 2010; West et al., 2013; Lopes-Bezerra et al., 2015). This lower content of cell wall mannan seems to be naturally compensated by increasing the β-glucan content. This is a well-documented compensatory mechanism driven by the activation of the calcineurin and the protein kinase C signaling pathways, where disruption of the synthesis of one cell wall component usually affects the levels of other components to avoid wall weakness (Bates et al., 2005, 2006; Mora-Montes et al., 2007; Walker et al., 2013). Our results also point out to subtle, but significant differences in the wall organization between members of the *C. parapsilosis* complex. In *C. orthopsilosis* all the β1,3-glucan and chitin is exposed in live cells, which suggest its cell wall proteins have even shorter mannans than *C. parapsilosis sensu stricto* and *C. metapsilosis*, but with more glycosylation sites occupied per protein. Alternatively, it could also be possible that mannans attached to *C. orthopsilosis* wall proteins are larger than those on the surface of other members of the complex, occupying less number of sites in the proteins, making the inner wall polysaccharides highly exposed at the cell surface. Nevertheless, further experiments are required to provide a conclusive explanation to these observations.

Results presented here clearly demonstrate that the human PMBCs-*C. parapsilosis sensu lato* interaction is different to that previously characterized in *C. albicans*. While yeast cells of *C. albicans* are a poor stimulus for cytokine production, the three members of the *C. parapsilosis* complex induced a strong dectin-1-dependent cytokine production. This is in line with the higher cell wall β1,3-glucan content in live *C. parapsilosis sensu lato*, but not in *C. albicans*. It is noteworthy to mention that results obtained here with *C. albicans* cells contrast with those previously reported (Netea et al., *2*006), where strong cytokine production was stimulated by yeast cells of this species. This apparent discrepancy relies in the fact that here we used live yeast cells, while in the previous work the main conclusions were based on HK organisms (Netea et al., 2006). When cells were heat killed we observed and increment in the chitin and the β1,3-glucan content, as previously reported (Gantner et al., 2005; Gow et al., 2007; Mora-Montes et al., 2011), and this correlated with a significant increment in cytokine production stimulated by *C. albicans*, *C. parapsilosis sensu stricto* and *C. metapsilosis*, stressing the importance of the dectin-1-β1,3-glucan interaction for a strong cytokine induction. In the case of *C. orthopsilosis*, we did not notice a significant increment in cytokine production upon heat killing, which support our result indicating most of the β1,3-glucan is naturally exposed on the *C. orthopsilosis* cell surface. Our results also suggest that *O*-linked mannans from yeast cells play a major role in the recognition of *C. parapsilosis sensu lato* than in *C. albicans*, where they are dispensable and redundant wall elements for cytokine stimulation by human PBMCs (Netea et al., 2006). It has been reported though, that *O*-linked mannans from *C. albicans* hypha have a major participation in cytokine stimulation by epithelial cells (Murciano et al., 2011), suggesting that the proposed redundant role during interaction with components of the immune system may be restricted to PBMCs. Here, we have evidence indicating that in the case of *C. parapsilosis sensu lato*, β-eliminated and HK cells stimulated similar cytokine levels, which suggest that upon removal of *O*-linked mannans, inner wall components are exposed and available to engage with PRRs, i.e., *O*-linked mannans have a role hiding inner wall components from immune receptors. Thus far, it has been reported that *N*-linked mannans mask the recognition of inner wall components and when disrupted, a significant cytokine production can be stimulated (Wheeler and Fink, 2006; Gow et al., 2007). Similar strategies, where a pro-inflammatory wall components is masked to avoid recognition by elements of the immune system, have been reported in *Histoplasma capsulatum* and *Aspergillus fumigatus*, where α-glucans and rodlet hydrophobins hide β1,3-glucan, respectively (Rappleye et al., 2007; Aimanianda et al., 2009). Therefore, it is feasible to conceive that in the case of *C. parapsilosis sensu lato*, both *N*- and *O*-linked mannans provide a disguising strategy to avoid recognition of β1,3-glucan by dectin-1. More experiments are required to demonstrate whether this structures are more abundant on the *C. parapsilosis sensu lato* cell wall or contain more mannose residues than those reported in *C. albicans*.

In this study we assessed the relevance of dectin-1, TLR4 and MR in the recognition of *C. parapsilosis sensu lato*. We chose these receptors because they are the main PRRs involved in the *C. albicans* immune recognition (Netea et al., 2002, 2006, 2015). TLR 4 is thus far the unique receptor for *C. albicans O*-linked mannans (Netea et al., 2006), and it is likely also participating in the recognition of the same cell wall component on *C. parapsilosis sensu lato*. The blocking experiments with either antibodies against receptors or laminarin, showed that TNFα stimulation by members of the *C. parapsilosis* complex, but not with *C. albicans* cells, depends on the engagement of TLR4, dectin-1 and MR with their ligands. These results contrast with our previous observation with β-eliminated cells, where TNFα levels upon disrupting the *O*-linked mannan-TLR4 interaction increased. The hypothesis to explain this apparent paradoxical response is that when the TLR4 receptor is blocked with anti-TLR4 antibodies, the *O*-linked mannans are still present on the surface of the fungal cell wall, hiding β1,3-glucan from the recognition by dectin-1. Interestingly, IL-6 production was only affected when human PBMCs were treated with anti-TLR4 antibodies and then stimulated with *C. orthopsilosis*, suggesting the *O*-linked mannans from this organism are different from those present on the surface of *C. parapsilosis sensu stricto* and *C. metapsilosis*. IL-10 production was strongly depend on engagement of dectin-1, as previously reported (Reid et al., 2009). It is noteworthy to mention that the IL-1β production by *C. parapsilosis sensu lato* was partially dependent on MR recognition, but not dectin-1 nor TLR4. This observation contrast with that reported earlier (Toth et al., 2013), where IL-1β stimulation by *C. parapsilosis sensu stricto* was dectin-1 dependent. Since the *C. albicans* immune recognition via dectin-1 is fungal strain-specific (Marakalala et al., 2013), it is feasible to conceive that this discrepancy can be explained because the genetic background of the strain used here (SZMC 8110) and in the previous report (GA-1) (Toth et al., 2013). In cells stimulated by *C. albicans*, IL-1β production is dependent on the activation of dectin-1, TLR2 and MR (van de Veerdonk et al., 2009); thus, different PRRs or downstream signaling components after *C. parapsilosis sensu lato* recognition could be responsible of this difference.

It has been previously demonstrated that cell wall preparations or non-purified wall components, such as zymosan, are potent inductors of cytokines (Brown et al., 2003; Netea et al., 2006); while purified wall components are unable to stimulated cytokine production (Dennehy et al., 2008; van de Veerdonk et al., 2009), and can block cytokine production when immune cells are stimulated (Maneu et al., 2011; Mora-Montes et al., 2011; Huang et al., 2012; Cohen-Kedar et al., 2014). These observations have been the base to propose the co-stimulatory theory, where a strong cytokine response involves the engagement of not only one but two or more PRRs at once (Netea et al., 2015). Although it is likely mannans from *C. albicans* and *C. parapsilosis sensu lato* are different, here it was demonstrated that purified *N*- and *O*-linked mannan preparations do not stimulate cytokine production and are capable to block proper *Candida* recognition, in a similar way that anti-MR and anti-TLR4 antibodies, respectively. Thus, they can be a reliable and alternative tool to assess the contribution of these PRRs during the innate immune recognition of fungal cells.

Overall, our data clearly demonstrate that the current knowledge about *C. albicans* biology and interaction with the host cannot be extrapolated to other members of the genus. *C. parapsilosis sensu lato* displays significant differences in the cell wall that impact the recognition by human PBMCs, stimulating stronger cytokine production, via the increased exposure of β1,3-glucan at the cell surface. Finally, our results suggest different contribution of dectin-1, MR and TLR4 in the recognition of the members of the *C. parapsilosis* complex, which has to be taken in consideration when analyzing the *C. parapsilosis sensu lato*-host interaction.

## Author Contributions

ML, AG, and HM-M conceived the study. EE-M, MN-A, LP-G, EM-M, and KC performed experiments. EE-M, MN-A, LP-G, EM-M, ML, KC, AG, and HM-M analyzed data. HM-M drafted the manuscript. EE-M, MN-A, LP-G, EM-M, ML, KC, AG, and HM-M revised and approved the manuscript.

## Conflict of Interest Statement

The authors declare that the research was conducted in the absence of any commercial or financial relationships that could be construed as a potential conflict of interest.

## References

[B1] Aimanianda V. Bayry J. Bozza S. Kniemeyer O. Perruccio K. Elluru S. R. (2009). Surface hydrophobin prevents immune recognition of airborne fungal spores. *Nature* 460 1117–1121. 10.1038/nature08264 19713928

[B2] Bates S. Hughes H. B. Munro C. A. Thomas W. P. MacCallum D. M. Bertram G. (2006). Outer chain N-glycans are required for cell wall integrity and virulence of *Candida albicans*. *J. Biol. Chem.* 281 90–98. 10.1074/jbc.M510360200 16263704

[B3] Bates S. MacCallum D. M. Bertram G. Munro C. A. Hughes H. B. Buurman E. T. (2005). *Candida albicans* Pmr1p, a secretory pathway P-type Ca2+/Mn2+-ATPase, is required for glycosylation and virulence. *J. Biol. Chem.* 280 23408–23415. 10.1074/jbc.M502162200 15843378

[B4] Brown G. D. Denning D. W. Gow N. A. Levitz S. M. Netea M. G. White T. C. (2012). Hidden killers: human fungal infections. *Sci. Transl. Med.* 4 165rv13. 10.1126/scitranslmed.3004404 23253612

[B5] Brown G. D. Herre J. Williams D. L. Willment J. A. Marshall A. S. Gordon S. (2003). Dectin-1 mediates the biological effects of beta-glucans. *J. Exp. Med.* 197 1119–1124. 10.1084/jem.20021890 12719478 PMC2193964

[B6] Butler G. Rasmussen M. D. Lin M. F. Santos M. A. Sakthikumar S. Munro C. A. (2009). Evolution of pathogenicity and sexual reproduction in eight *Candida* genomes. *Nature* 459 657–662. 10.1038/nature08064 19465905 PMC2834264

[B7] Cambi A. Netea M. G. Mora-Montes H. M. Gow N. A. Hato S. V. Lowman D. W. (2008). Dendritic cell interaction with *Candida albicans* critically depends on N-linked mannan. *J. Biol. Chem.* 283 20590–20599. 10.1074/jbc.M709334200 18482990 PMC2459306

[B8] Cohen-Kedar S. Baram L. Elad H. Brazowski E. Guzner-Gur H. Dotan I. (2014). Human intestinal epithelial cells respond to beta-glucans via Dectin-1 and Syk. *Eur. J. Immunol.* 44 3729–3740. 10.1002/eji.201444876 25251945

[B9] De Nobel J. G. Klis F. M. Munnik T. Priem J. Van Den Ende H. (1990). An assay of relative cell wall porosity in *Saccharomyces cerevisiae*, *Kluyveromyces lactis* and *Schizosaccharomyces pombe*. *Yeast* 6 483–490. 10.1002/yea.320060605 2080665

[B10] Dennehy K. M. Ferwerda G. Faro-Trindade I. Pyz E. Willment J. A. Taylor P. R. (2008). Syk kinase is required for collaborative cytokine production induced through Dectin-1 and Toll-like receptors. *Eur. J. Immunol.* 38 500–506. 10.1002/eji.200737741 18200499 PMC2430329

[B11] Diaz-Jimenez D. F. Mora-Montes H. M. Hernandez-Cervantes A. Luna-Arias J. P. Gow N. A. Flores-Carreon A. (2012). Biochemical characterization of recombinant *Candida albicans* mannosyltransferases Mnt1, Mnt2 and Mnt5 reveals new functions in O- and N-mannan biosynthesis. *Biochem. Biophys. Res. Commun.* 419 77–82. 10.1016/j.bbrc.2012.01.131 22326920 PMC3480643

[B12] Díaz-Jiménez D. Pérez-García L. Martínez-Álvarez J. Mora-Montes H. (2012). Role of the fungal cell wall in pathogenesis and antifungal resistance. *Curr. Fungal Infect. Rep.* 6 275–282. 10.1007/s12281-012-0109-7

[B13] Endres S. Ghorbani R. Lonnemann G. van der Meer J. W. Dinarello C. A. (1988). Measurement of immunoreactive interleukin-1 beta from human mononuclear cells: optimization of recovery, intrasubject consistency, and comparison with interleukin-1 alpha and tumor necrosis factor. *Clin. Immunol. Immunopathol.* 49 424–438. 10.1016/0090-1229(88)90130-4 2461270

[B14] Gago S. Garcia-Rodas R. Cuesta I. Mellado E. Alastruey-Izquierdo A. (2014). *Candida parapsilosis*, *Candida orthopsilosis*, and *Candida metapsilosis* virulence in the non-conventional host *Galleria mellonella*. *Virulence* 5 278–285. 10.4161/viru.26973 24193303 PMC3956503

[B15] Gantner B. N. Simmons R. M. Underhill D. M. (2005). Dectin-1 mediates macrophage recognition of *Candida albicans* yeast but not filaments. *EMBO J.* 24 1277–1286. 10.1038/sj.emboj.7600594 15729357 PMC556398

[B16] Gillum A. M. Tsay E. Y. Kirsch D. R. (1984). Isolation of the *Candida albicans* gene for orotidine-5′-phosphate decarboxylase by complementation of *S. cerevisiae* ura3 and *E. coli* pyrF mutations. *Mol. Gen. Genet.* 198 179–182. 10.1007/BF00328721 6394964

[B17] Gow N. A. R. Netea M. G. Munro C. A. Ferwerda G. Bates S. Mora-Montes H. M. (2007). Immune recognition of *Candida albicans* beta-glucan by dectin-1. *J. Infect. Dis.* 196 1565–1571. 10.1086/523110 18008237 PMC2655640

[B18] Graham L. M. Tsoni S. V. Willment J. A. Williams D. L. Taylor P. R. Gordon S. (2006). Soluble Dectin-1 as a tool to detect beta-glucans. *J. Immunol. Methods* 314 164–169. 10.1016/j.jim.2006.05.013 16844139

[B19] Heinsbroek S. E. Taylor P. R. Martinez F. O. Martinez-Pomares L. Brown G. D. Gordon S. (2008). Stage-specific sampling by pattern recognition receptors during *Candida albicans* phagocytosis. *PLoS Pathog.* 4:e1000218 10.1371/journal.ppat.1000218 PMC2583056 19043561

[B20] Hobson R. P. Munro C. A. Bates S. MacCallum D. M. Cutler J. E. Heinsbroek S. E. (2004). Loss of cell wall mannosylphosphate in *Candida albicans* does not influence macrophage recognition. *J. Biol. Chem.* 279 39628–39635. 10.1074/jbc.M405003200 15271989

[B21] Huang H. Ostroff G. R. Lee C. K. Agarwal S. Ram S. Rice P. A. (2012). Relative contributions of dectin-1 and complement to immune responses to particulate beta-glucans. *J. Immunol.* 189 312–317. 10.4049/jimmunol.1200603 22649195 PMC3381926

[B22] Linden J. R. Kunkel D. Laforce-Nesbitt S. S. Bliss J. M. (2013). The role of galectin-3 in phagocytosis of *Candida albicans* and *Candida parapsilosis* by human neutrophils. *Cell. Microbiol.* 15 1127–1142. 10.1111/cmi.12103 23279221 PMC3640745

[B23] Lopes-Bezerra L. M. Lozoya-Perez N. E. Lopez-Ramirez L. A. Martinez-Alvarez J. A. Teixeira M. M. Felipe M. S. (2015). Functional characterization of *Sporothrix schenckii* glycosidases involved in the N-linked glycosylation pathway. *Med. Mycol.* 53 60–68. 10.1093/mmy/myu057 25526779

[B24] Maneu V. Yanez A. Murciano C. Molina A. Gil M. L. Gozalbo D. (2011). Dectin-1 mediates in vitro phagocytosis of *Candida albicans* yeast cells by retinal microglia. *FEMS Immunol. Med. Microbiol.* 63 148–150. 10.1111/j.1574-695X.2011.00829.x 21668824

[B25] Marakalala M. J. Vautier S. Potrykus J. Walker L. A. Shepardson K. M. Hopke A. (2013). Differential adaptation of *Candida albicans* in vivo modulates immune recognition by dectin-1. *PLoS Pathog.* 9:e1003315 10.1371/journal.ppat.1003315 PMC3630191 23637604

[B26] McKenzie C. G. Koser U. Lewis L. E. Bain J. M. Mora-Montes H. M. Barker R. N. (2010). Contribution of *Candida albicans* cell wall components to recognition by and escape from murine macrophages. *Infect. Immun.* 78 1650–1658. 10.1128/IAI.00001-10 20123707 PMC2849426

[B27] Miyake K. (2007). Innate immune sensing of pathogens and danger signals by cell surface Toll-like receptors. *Semin. Immunol.* 19 3–10. 10.1016/j.smim.2006.12.002 17275324

[B28] Mora-Montes H. M. Bates S. Netea M. G. Castillo L. Brand A. Buurman E. T. (2010). A multifunctional mannosyltransferase family in *Candida albicans* determines cell wall mannan structure and host-fungus interactions. *J. Biol. Chem.* 285 12087–12095. 10.1074/jbc.M109.081513 20164191 PMC2852947

[B29] Mora-Montes H. M. Bates S. Netea M. G. Diaz-Jimenez D. F. Lopez-Romero E. Zinker S. (2007). Endoplasmic reticulum alpha-glycosidases of *Candida albicans* are required for N glycosylation, cell wall integrity, and normal host-fungus interaction. *Eukaryot. Cell* 6 2184–2193. 10.1128/EC.00350-07 17933909 PMC2168260

[B30] Mora-Montes H. M. McKenzie C. Bain J. M. Lewis L. E. Erwig L. P. Gow N. A. (2012). Interactions between macrophages and cell wall oligosaccharides of *Candida albicans*. *Methods Mol. Biol.* 845 247–260. 10.1007/978-1-61779-539-8_16 22328379

[B31] Mora-Montes H. M. Netea M. G. Ferwerda G. Lenardon M. D. Brown G. D. Mistry A. R. (2011). Recognition and blocking of innate immunity cells by *Candida albicans* chitin. *Infect. Immun.* 79 1961–1970. 10.1128/IAI.01282-10 21357722 PMC3088140

[B32] Murciano C. Moyes D. L. Runglall M. Islam A. Mille C. Fradin C. (2011). *Candida albicans* cell wall glycosylation may be indirectly required for activation of epithelial cell proinflammatory responses. *Infect. Immun.* 79 4902–4911. 10.1128/IAI.05591-11 21930756 PMC3232641

[B33] Nemeth T. Toth A. Szenzenstein J. Horvath P. Nosanchuk J. D. Grozer Z. (2013). Characterization of virulence properties in the *C. parapsilosis* sensu lato species. *PLoS ONE* 8:e68704 10.1371/journal.pone.0068704 PMC3706360 23874732

[B34] Netea M. G. Gow N. A. Munro C. A. Bates S. Collins C. Ferwerda G. (2006). Immune sensing of *Candida albicans* requires cooperative recognition of mannans and glucans by lectin and Toll-like receptors. *J. Clin. Invest.* 116 1642–1650. 10.1172/JCI27114 16710478 PMC1462942

[B35] Netea M. G. Joosten L. A. van der Meer J. W. Kullberg B. J. van de Veerdonk F. L. (2015). Immune defence against *Candida* fungal infections. *Nat. Rev. Immunol.* 15 630–642. 10.1038/nri3897 26388329

[B36] Netea M. G. Van Der Graaf C. A. Vonk A. G. Verschueren I. Van Der Meer J. W. Kullberg B. J. (2002). The role of toll-like receptor (TLR) 2 and TLR4 in the host defense against disseminated candidiasis. *J. Infect. Dis.* 185 1483–1489. 10.1086/340511 11992285

[B37] Pammi M. Holland L. Butler G. Gacser A. Bliss J. M. (2013). *Candida parapsilosis* is a significant neonatal pathogen: a systematic review and meta-analysis. *Pediatr. Infect. Dis. J.* 32 e206–e216. 10.1097/INF.0b013e3182863a1c 23340551 PMC3681839

[B38] Rappleye C. A. Eissenberg L. G. Goldman W. E. (2007). Histoplasma capsulatum alpha-(1,3)-glucan blocks innate immune recognition by the beta-glucan receptor. *Proc. Natl. Acad. Sci. U.S.A.* 104 1366–1370. 10.1073/pnas.0609848104 17227865 PMC1783108

[B39] Reid D. M. Gow N. A. R. Brown G. D. (2009). Pattern recognition: recent insights from Dectin-1. *Curr. Opin. Immunol.* 21 30–37. 10.1016/j.coi.2009.01.003 19223162 PMC2684021

[B40] Saijo S. Iwakura Y. (2011). Dectin-1 and Dectin-2 in innate immunity against fungi. *Int. Immunol.* 23 467–472. 10.1093/intimm/dxr046 21677049

[B41] Schwartz S. N. Medoff G. Kobayashi G. S. Kwan C. N. Schlessinger D. (1972). Antifungal properties of polymyxin B and its potentiation of tetracycline as an antifungal agent. *Antimicrob. Agents Chemother.* 2 36–40. 10.1128/AAC.2.1.36 4364558 PMC444262

[B42] Shibata N. Ikuta K. Imai T. Satoh Y. Satoh R. Suzuki A. (1995). Existence of branched side chains in the cell wall mannan of pathogenic yeast, *Candida albicans*. Structure-antigenicity relationship between the cell wall mannans of *Candida albicans* and *Candida parapsilosis*. *J. Biol. Chem.* 270 1113–1122. 10.1074/jbc.270.3.1113 7836369

[B43] Spreghini E. Orlando F. Tavanti A. Senesi S. Giannini D. Manso E. (2012). In vitro and in vivo effects of echinocandins against *Candida parapsilosis* sensu stricto, *Candida orthopsilosis* and *Candida metapsilosis*. *J. Antimicrob. Chemother.* 67 2195–2202. 10.1093/jac/dks180 22635526

[B44] Szenzenstein J. Gacser A. Grozer Z. Farkas Z. Nagy K. Vagvolgyi C. (2013). Differential sensitivity of the species of *Candida parapsilosis* sensu lato complex against statins. *Mycopathologia* 176 211–217. 10.1007/s11046-013-9689-1 23943427

[B45] Tavanti A. Davidson A. D. Gow N. A. Maiden M. C. Odds F. C. (2005). *Candida orthopsilosis* and *Candida metapsilosis* spp. nov. to replace *Candida parapsilosis* groups II and III. *J. Clin. Microbiol.* 43 284–292. 10.1128/JCM.43.1.284-292.2005 15634984 PMC540126

[B46] Toth A. Csonka K. Jacobs C. Vagvolgyi C. Nosanchuk J. D. Netea M. G. (2013). *Candida albicans* and *Candida parapsilosis* induce different T-cell responses in human peripheral blood mononuclear cells. *J. Infect. Dis.* 208 690–698. 10.1093/infdis/jit188 23661798 PMC3719900

[B47] Trevino-Rangel Rde J. Gonzalez J. G. Gonzalez G. M. (2013). Aspartyl proteinase, phospholipase, esterase and hemolysin activities of clinical isolates of the *Candida parapsilosis* species complex. *Med. Mycol.* 51 331–335. 10.3109/13693786.2012.712724 22928925

[B48] Trofa D. Gacser A. Nosanchuk J. D. (2008). *Candida parapsilosis*, an emerging fungal pathogen. *Clin. Microbiol. Rev.* 21 606–625. 10.1128/CMR.00013-08 18854483 PMC2570155

[B49] van de Veerdonk F. L. Joosten L. A. Devesa I. Mora-Montes H. M. Kanneganti T. D. Dinarello C. A. (2009). Bypassing pathogen-induced inflammasome activation for the regulation of interleukin-1beta production by the fungal pathogen *Candida albicans*. *J. Infect. Dis.* 199 1087–1096. 10.1086/597274 19222370

[B50] Walker L. A. Gow N. A. Munro C. A. (2013). Elevated chitin content reduces the susceptibility of *Candida* species to caspofungin. *Antimicrob. Agents Chemother.* 57 146–154. 10.1128/AAC.01486-12 23089748 PMC3535899

[B51] West L. Lowman D. W. Mora-Montes H. M. Grubb S. Murdoch C. Thornhill M. H. (2013). Differential virulence of *Candida glabrata* glycosylation mutants. *J. Biol. Chem.* 288 22006–22018. 10.1074/jbc.M113.478743 23720756 PMC3724654

[B52] Wheeler R. T. Fink G. R. (2006). A drug-sensitive genetic network masks fungi from the immune system. *PLoS Pathog.* 2:e35 10.1371/journal.ppat.0020035 PMC1447670 16652171

